# Sensing Inflammation Biomarkers with Electrolyte‐Gated Organic Electronic Transistors

**DOI:** 10.1002/adhm.202100955

**Published:** 2021-08-22

**Authors:** Bernhard Burtscher, Pamela Allison Manco Urbina, Chiara Diacci, Simone Borghi, Marcello Pinti, Andrea Cossarizza, Carlo Salvarani, Magnus Berggren, Fabio Biscarini, Daniel T. Simon, Carlo A. Bortolotti

**Affiliations:** ^1^ Laboratory of Organic Electronics Department of Science and Technology Linköping University Norrköping 60174 Sweden; ^2^ Department of Life Sciences University of Modena and Reggio Emilia Via Campi 103 Modena 41125 Italy; ^3^ Department of Medical and Surgical Sciences for Children and Adults University of Modena and Reggio Emilia Via Campi 287 Modena 41125 Italy; ^4^ Rheumatology Unit University of Modena and Reggio Emilia Medical School Azienda Ospedaliero‐Universitaria Policlinico di Modena Modena 41124 Italy; ^5^ Center for Translation Neurophysiology Istituto Italiano di Tecnologia Via Fossato di Mortara 17–19 Ferrara 44100 Italy

**Keywords:** biosensors, cytokines, EGOT, inflammation, organic bioelectronics

## Abstract

An overview of cytokine biosensing is provided, with a focus on the opportunities provided by organic electronic platforms for monitoring these inflammation biomarkers which manifest at ultralow concentration levels in physiopathological conditions. Specifically, two of the field's state‐of‐the‐art technologies—organic electrochemical transistors (OECTs) and electrolyte gated organic field effect transistors (EGOFETs)—and their use in sensing cytokines and other proteins associated with inflammation are a particular focus. The overview will include an introduction to current clinical and “gold standard” quantification techniques and their limitations in terms of cost, time, and required infrastructure. A critical review of recent progress with OECT‐ and EGOFET‐based protein biosensors is presented, alongside a discussion onthe future of these technologies in the years and decades ahead. This is especially timely as the world grapples with limited healthcare diagnostics during the Coronavirus disease (COVID‐19)pandemic where one of the worst‐case scenarios for patients is the “cytokine storm.” Clearly, low‐cost point‐of‐care technologies provided by OECTs and EGOFETs can ease the global burden on healthcare systems and support professionals by providing unprecedented wealth of data that can help to monitor disease progression in real time.

## Graphical abstract

The roles, significance, and potential diagnostic capabilities of cytokines, the body's endogenous inflammation biomarkers are introduced. The report proceeds with a focus on cytokine biosensors based on organic electrochemical transistors and electrolyte gated organic field effect transistors and concludes with an outlook on the benefits of such organic electronic technologies.

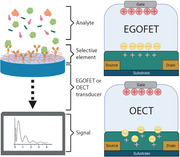

## 1 Introduction

Complex organisms, from plants to primates, require a well‐developed specific protective capacity to counteract noxious stimuli such as pathogen invasion, tissue damage,^[^ ^1^, ^2^ ^]^ and other kinds of hazard.^[^ ^3^ ^]^ The initial host response triggered by these stimuli in vertebrates is inflammation, which is a localized dynamic process involving blood components, more specifically leukocytes and plasma proteins, together with tissue cells. Its purpose is not only to eliminate the harmful agents but also to facilitate repairing processes.

**Figure 1:**
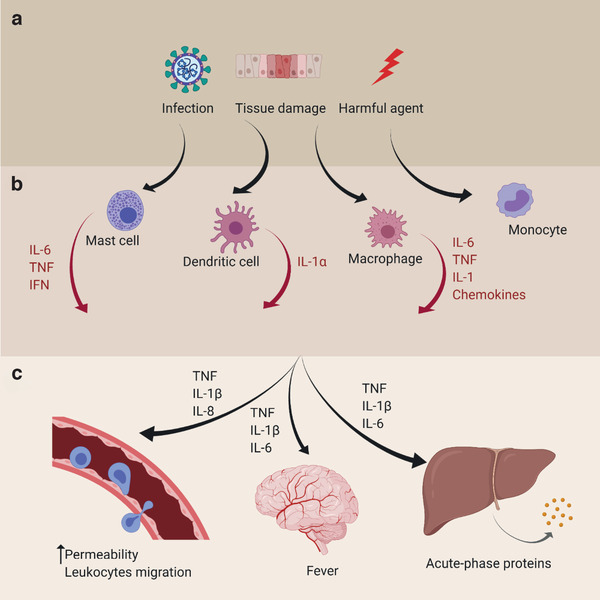
Inflammatory pathways. a) A pathological, physical, chemical, or other harmful trigger initiates a response from the body's immune system, b) comprising mast cells, dendritic cells, macrophages, and monocytes, among others. These immune cells release immune response compounds (cytokines, chemokines) which have (c) downstream effects on, e.g., the vascular system, fever response, or organ function.

The inflammatory process involves four components: i) inflammatory inducers, the aforementioned harmful agents that trigger the inflammatory response; ii) sensors, such as macrophages, mast cells, and dendritic cells, which stimulate the production of chemical mediators in response to inducers; iii) mediators, among them cytokines, which are cell‐to‐cell messengers that primarily act locally at the target tissue with very fast response; and iv) target tissues, where mediators elicit the inflammatory response until neutralizing the noxious stimuli.^[^ ^2^, ^3^, ^4^ ^]^ The interplay between the main actors in inflammation is illustrated in **Figure** **1**. The inflammatory response takes place within minutes, and can last as long as several days, until the noxious condition has been eliminated and the tissue has been repaired. This process is known as the acute inflammatory response, which is controlled and self‐limited through the activation of anti‐inflammatory mechanisms in order to maintain tissue homeostasis.

When the acute inflammatory response fails and noxious stimuli remain, inflammation persists, and becomes chronic. This condition lasts from days to years, and can be local, confined to the site of the acute response, or systemic, affecting large parts of (or the entire) body.^[^ ^3^, ^5^ ^]^ Chronic inflammation is associated with a wide range of pathologies, including atherosclerosis, cancer,^[^ ^6^ ^]^ neurodegenerative diseases,^[^ ^7^, ^8^ ^]^ and obesity,^[^ ^9^, ^10^ ^]^ while acute inflammation is associated with sepsis, human immunodeficiency virus (HIV) infection,^[^ ^11^ ^]^ and many others, including Coronavirus disease (COVID‐19).^[^ ^2^, ^4^, ^12^ ^]^ Systemic chronic low‐grade inflammation is characterized by a subclinical increase in inflammatory parameters and has been linked to specific social, environmental, and lifestyle factors, such as exposure to industrial toxins, high‐calorie diet, and physical inactivity, among others. However, this condition can be observed even in the absence of evident signs and symptoms of inflammation, for instance, in elderly subjects experiencing “successful aging” (aging without comorbidities).^[^ ^13^, ^14^, ^15^ ^]^ Consequently, significant efforts have been devoted to investigating the correlation between the onset of different pathologies and inflammation‐related biomarkers, among which acute‐phase proteins (e.g., C‐reactive protein, CRP) and cytokines are possibly the most relevant. High levels of inflammatory biomarkers have been associated with disease risk, progression, and adverse prognosis. Although some molecules, such as CRP, are routinely used in clinical settings for diagnostic purposes, it is worth mentioning the importance of the analysis of several biomarkers, ideally simultaneously, in order to obtain a characteristic disease “fingerprint” as well as to improve disease prognosis, since a set of biomarkers can give more accurate information than a single one.^[^ ^8^, ^12^ ^]^

Chemical mediators of inflammation include cytokines, a heterogenous group of polypeptides, proteins, and glycoproteins, which mediate cell signaling and communication among cells of the immune system as well as between immune cells and other tissues and organs. Cytokines comprise monomers, dimers, or trimers, as in the case for tumor necrosis factor‐alpha (TNF‐*α*). Briefly, in response to noxious stimuli, a subset of cytokines called proinflammatory cytokines is secreted by different cell types, e.g., activated monocytes/macrophages, T helper cells, and mast cells, in order to elicit inflammation and to recruit and activate innate immune cells in the tissue of residence. When secreted in low amounts, proinflammatory cytokines act locally. If secreted in much larger amounts, cytokine “spillover” from inflamed tissue to the circulatory system can occur. As a consequence, the levels of circulating cytokines, which lie in the picomolar range under physiological conditions, are increased by up to a 1000‐fold during the inflammatory response.^[^ ^3^, ^16^, ^17^ ^]^

A given cytokine function is heavily dependent on both the target tissue and the stimuli and therefore each cytokine can be involved in a wide range of pathways that lead to diverse effects due to their multiple biological properties. This feature is known as pleiotropy and is a hallmark of cytokines.^[^ ^17^ ^]^ Therefore, cytokine upregulation has been associated with many diseases, both as a cause and as an effect. This is one of the primary reasons why cytokines have received so much attention in recent years for disease diagnosis, prognosis, and therapy monitoring for a range of pathologies. In addition to the general diseases related to inflammation (mentioned above), pathologies specifically associated with cytokine upregulation include atherosclerosis,^[^ ^18^ ^]^ heart failure,^[^ ^19^ ^]^ cancer,^[^ ^20^ ^]^ rheumatoid arthritis,^[^ ^21^ ^]^ psoriasis, and systemic sclerosis.^[^ ^22^ ^]^

Another hallmark of cytokines is how they work as a network of interacting compounds in a concerted immune response.^[^ ^16^ ^]^ A recent computational model, immuneXpresso (iX), was built to perform a meta‐analysis in order to obtain an immune profile associated with each disease including the specific cytokine profile.^[^ ^23^ ^]^ The software analyzed literature in PubMed, by surveying the intercellular interactions involving cytokines and their association with disease development. The study identified a subset of cytokines that regularly appeared as highly active in the network, including TNF‐*α*, IL‐6, tumor growth factor‐*β* (TGF‐*β*), and interferon‐*γ* (IFN‐*γ*). Additional analysis of cytokine profiles in 188 diseases resulted in a group of “backbone cytokines” which were found to be common to most of the diseases in the study. These findings highlight the importance of using cytokine profiling, rather than individual cytokines, as inflammatory‐associated disease biomarkers that could be used to monitor a wide array of diseases.

With the recent coronavirus pandemic, the “cytokine storm”—the sudden uncontrolled secretion of several kinds of cytokines in response to infectious and noninfectious diseases—has entered the public discourse.^[^ ^16^, ^24^ ^]^ In the cytokine storm, the inflammation moves from local to systemic via the circulation, resulting in a high increase of cytokine levels in the blood (i.e., spillover). It has been observed that some people are more susceptible to the cytokine storm than others, developing a more severe clinical condition, although the molecular mechanism underlying this difference is not yet clear. Cytokine storm syndrome could even mimic sepsis symptomatology, leading to death in severe cases. The term “cytokine storm” started to appear more frequently in scientific reports from the early 2000s, especially in relation to viral diseases including the severe acute respiratory syndrome coronavirus (SARS‐CoV) infection.^[^ ^24^ ^]^ Studies performed in the years following the SARS epidemic (2002), showed the role of cytokines in the pathogenesis—especially in the critical phase of the disease—in which severe symptoms seemed to be associated with a cytokine storm. This symptomatology corresponded to lung damage with rupture of pulmonary alveoli causing inefficient oxygenation, and thus the “acute respiratory distress syndrome,” all as a consequence of the excessive release of cytokines.^[^ ^25^, ^26^ ^]^ The recent pandemic situation of COVID‐19, caused by the coronavirus SARS‐Cov‐2, has rekindled attention on the important role of cytokines in the evolution of the disease, and highlighted the use of inflammatory cytokines as disease biomarkers. Several studies have found a strong correlation between unfavorable prognosis of COVID‐19 with high levels of inflammatory cytokines,^[^ ^27^ ^]^ especially with IL‐6 and CRP.^[^ ^28^, ^29^, ^30^, ^31^, ^32^ ^]^ Furthermore, taking into consideration the short half‐life of cytokines, and that some diseases display an acute onset (as with COVID‐19), development of fast, accurate, and reliable cytokine biosensors has emerged as an imperative need for point‐of‐care (PoC) testing.^[^ ^33^ ^]^

During the last couple of decades, the number of studies focusing on cytokines as disease biomarkers has increased considerably, thus driving significant advances in various biosensing technologies. Those biosensors are needed not only for diagnosis but also for monitoring the treatment of diseases. The above‐mentioned features define the necessary requisites and figures‐of‐merit for cytokine biosensors: i) ultralow limit of detection (LOD) and high sensitivity, since cytokines are present at very low concentrations (sub‐pm) under physiological conditions; ii) wide dynamic range, spanning pm to nm concentrations and beyond, as cytokines levels can reach a 1000‐fold increase under pathological conditions; iii) simultaneous detection of a set of cytokines, to monitor changes in the expression at a network level, and allowing for detection of altered balance between pro‐ and anti‐inflammatory cytokines; iv) fast response, especially in those cases where an acute onset is observed, and v) easy implementation at the PoC for disease diagnosis and monitoring. In addition, manufacturing biosensors with features such as low power consumptions, ease of use, real‐time read‐out, low‐cost, and disposability would be beneficial for large‐scale implementation and distribution. In the current COVID‐19 pandemic, reliable biosensors for screening positive cases have come to the forefront as a potentially game‐changing tool to fight the spread of the virus. Biosensors with these characteristics will be useful, not only in this current situation, but also in future outbreaks, and are thus now one of the major focuses of the research community.

## 2 State‐of‐the‐Art in Cytokine Sensing

**Figure 2:**
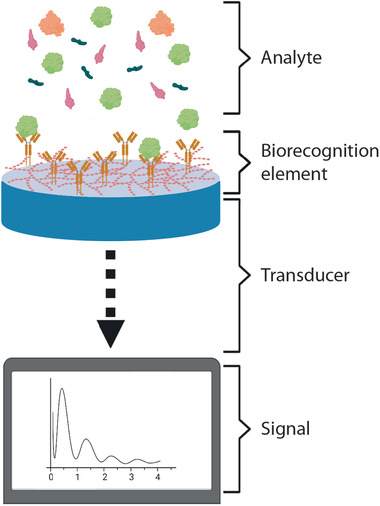
Schematics of a label‐free biosensor.

Biosensors are devices for specific analyte detection, providing an output signal that correlates to analyte concentration. A biosensor is composed by: i) a biorecognition element (typically an antibody, aptamer, or an enzyme), which detects and binds specifically the analyte; ii) a transducer that converts a physical or chemical change (typically, a binding event or an enzymatic reaction) into a measurable signal (the main transducers being electrochemical, optical, piezoelectric, thermometric, and magnetic); iii) the read‐out, which is the processed and displayed signal (**Figure** **2**).^[^ ^34^, ^35^ ^]^ Clearly, both the specific biorecognition element and the transducing strategy affect the biosensor figures of merit, selectivity, sensitivity, and dynamic range of detection above all.

Antibodies (Abs) have been (and still are) by far the most widely employed biorecognition element in biosensing. Nevertheless, in the last two decades significant efforts have been put to seek for alternative binders, in order to overcome the main limitations connected with the use of Abs, namely their complex synthesis procedure, the unknown sequence and their large dimensions, which might represent a severe limitation in some electrochemical or electrical sensing schemes, as the binding event would take place well beyond the Debye length, thus hampering the device sensitivity. Nonimmunoglobulin‐based scaffold are emerging as substitutes for the Abs in both theragnostic and diagnostics. They can be subdivided in DNA/RNA aptamers and peptide aptamers. The former are synthetic oligonucleotides, specifically generated and selected in vitro through a process called systemic evolution of ligands by exponential enrichment.^[^ ^36^ ^]^ Peptide aptamers are based on relatively small, structurally robust proteins as scaffold (like proteases), with connecting loops that make them highly selective for protein recognition.^[^ ^37^, ^38^ ^]^ These engineered scaffolds present high affinity and specificity, low‐cost production, and small size, which also confers them long‐term storage stability. Moreover, the high control on their sequence and the facile synthesis allow the design of ad hoc modifications, such as the insertion of linkers or functional groups, that would enable controlled immobilization on a surface.^[^ ^39^ ^]^ As will be clear from the next sections, both immunoglobulin‐ and nonimmunoglobulin‐based binders have been employed for label free cytokine detection.

Enzyme‐linked immunosorbent assay (ELISA) is a quantitative biochemical technique which relies on antibody‐antigen interaction and colorimetric reaction to measure specific analyte. This assay remains the “gold standard” technology for cytokines detection and quantification, with a very high sensitivity in the sub pg mL^−1^ range of concentration for cytokines such as IL‐6 (LOD of 0.09 pg mL^−1^).^[^ ^40^ ^]^ ELISA is used for detection of a single analyte; however, as highlighted in the previous section, when it comes to cytokines, a simultaneous detection of multiple biomarkers is preferred. Flow cytometry‐based technology (e.g., Luminex) exploits laser beam and fluorescent labeled Abs flowing down a chamber for the detection of biomarkers and cells. This technique has emerged to overcome the limitation of detecting single molecule, being widely used in research and clinics with similar sensitivity as ELISA, detecting on the sub pg mL^−1^ range of concentration (LOD of 0.2 pg mL^−1^ for IL‐6).^[^ ^41^ ^]^ Both commercial techniques are highly sensitive and selective, but they present some limitations: first of all, both are time consuming, the whole process taking 3–8 h.^[^ ^42^ ^]^ ELISA requires relatively larger sample volumes (50–100 µL per sample), while Luminex requires only 25–50 µL for multiple samples, but both need specialized equipment and personnel, as well as fluorescent labeling for detection. Therefore, efforts have been put to overcome all these limitations, in search for a technique that could also be implemented at the PoC, facilitating the transition from the benchtop to the clinics. Novel biosensing strategies have been developed during the last 20 years, and have been widely reviewed.^[^ ^42^, ^43^, ^44^, ^45^ ^]^ The scope of this section is to provide an overview of the most recent (i.e., within the last 5 years) advances in cytokine detection, with a focus on label‐free techniques. In particular, for this progress report, we define as “labels” foreign molecules that need to be added prior or after the incubation of the analyte with the sensing unit. In what follows, we classify and briefly describe the most recently demonstrated label free cytokine biosensors classified based on the transducer principle and close the present section with some comments regarding the necessary requisites that novel sensing platforms should feature to fill the gap between laboratory‐scale research and implementation at the PoC.

### 2.1 Optical Biosensors

Label free devices based on optical transduction exploit the interaction between light and the sensing element immobilized on the surface. When the biorecognition unit binds the analyte, one of the properties (e.g., phase or intensity) of the light, that is reflected at the sensing interface, changes in a way that is proportional to the amount of adsorbed analyte. Surface plasmon resonance (SPR) is one of the most established label free optical methods, the typical setup of which is a thin film of gold on a glass prism, with the receptors bound to the gold surface at the interface with a solution flowing on top of it and containing the analyte. Surface plasmons are collective oscillations of free electron density at the surface of a metal. SPR occurs when surface plasmons are excited by polarized light at the angle of total internal reflection, generating an evanescent wave.^[^ ^46^ ^]^ The angle of total internal reflection is dependent on the refractive index of the medium at the metal/solution interface within up to 300 nm far from the surface (corresponding to the penetration of the evanescent field into the dielectric) and can therefore be affected by binding of the target to the surface‐immobilized binders (Abs, aptamers) and more generally by the amount of mass adsorbed on the surface.^[^ ^47^ ^]^

**Figure 3:**
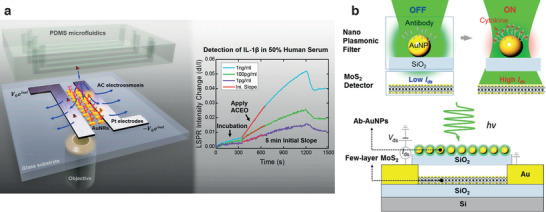
Optical based devices for cytokines sensors. a) Nanoplasmonic device integrated with microfluidics with an electroosmotic effect to achieve fast and sensitive detection. Reproduced with permission.^[^ ^54^ ^]^ Copyright 2017, American Chemical Society. b) Nanoplasmonic fiber for cytokine detection, based on an electronic readout signal. Reproduced with permission.^[^ ^55^ ^]^ Copyright 2017, American Chemical Society.

In recent years, optical cytokine biosensors were demonstrated based on plasmonic nanobiosensors, which rely on localized surface plasmon resonance (LSPR) that takes place when conduction band electrons of nanometer‐sized objects (e.g., a nanoparticle, whose dimensions are comparable to the wavelength of light) participate in the collective oscillations. Excellent reviews on LSPR‐based biosensors have been published.^[^ ^48^, ^49^ ^]^ Nanobiosensors take advantage of the dramatic improvements in nanofabrication of the last decades to overcome some of the limitations imposed by traditional SPR, such as the complex prism‐coupling instrumentation, which hampers multiplexing and miniaturization, and the fact that penetration depth of the evanescent wave is much larger than average size of target molecules, thus affecting the sensitivity of traditional SPR.^[^ ^50^ ^]^ As cytokines are relatively small proteins, nanophotonic biosensors have proven to be highly effective in their detection, allowing detection of multiple cytokines on a microarray chip functionalized with different Abs^[^ ^42^ ^]^ and enabling the integration of cell culture modules to achieve real‐time analysis (in particular, detection of vascular endothelial growth factor (VEGF) of live cancer cells^[^ ^51^ ^]^) and even reaching down to monitoring of IL‐2 secretion from a single cell.^[^ ^51^, ^52^, ^53^ ^]^ Further recent examples of nanostructured cytokine assays involve the integration of optical and electrical components. This is the case of the IL‐1*β* biosensor composed by nanoplasmonic unit integrated with microelectrodes and microfluidics developed by Song et al. The electrodes present in the sensor are producing an electroosmotic effect in the electrolyte: the electrohydrodynamic agitation enhances the performance of the sensor both in terms of response time (5–15 min) and high sensitivity (1 pg mL^−1^) of cytokine detection in diluted human serum (**Figure** **3**a).^[^ ^54^ ^]^ Cai et al. integrated molybdenum disulfide (MoS_2_) photoconductive component with a nanoplasmonic optical filter that tunes the incident light delivered to the photoconductive layer through the binding of the analyte (IL‐1*β*) to anti IL‐1*β*‐conjugated gold nanoparticles thus inducing LSPR shifts (Figure 3b).^[^ ^55^ ^]^ Other cytokine sensors based on optical transducers are taking advantage of the quantum dots properties, connected with aptamers for the detection of TNF‐*α*.^[^ ^56^ ^]^

### 2.2 Electrochemical Biosensors

These devices measure changes of an electrical signal due to the binding event of the analyte to the corresponding biorecognition element. The electrical signal can be processed as different measured parameters such as current, potential, or impedance.^[^ ^34^ ^]^ Cytokines are not electrochemically active: therefore, they can be indirectly quantified using electrochemical means by monitoring the change in the response of a redox probe in solution (typically, potassium ferri/ferrocyanide couple) upon binding of the target to the working electrode modified with a specific binder (for a comprehensive overview of cytokine detection with electrochemical biosensors see review from Dutta et al.^[^ ^57^ ^]^). Electrochemical impedance spectroscopy (EIS) is by far the most widely employed electrochemical method for cytokine and chemokine detection: EIS‐based biosensors have been demonstrated for IL‐6,^[^ ^58^, ^59^ ^]^ IL‐8,^[^ ^60^, ^61^ ^]^ IL1*α*,^[^ ^62^, ^63^ ^]^ TNF‐*α*,^[^ ^64^, ^65^ ^]^ IFN‐*γ*,^[^ ^66^, ^67^ ^]^ and TGF‐*β*.^[^ ^68^ ^]^ Within this pool of biosensors, one can identify at least three different materials strategies that have been pursued to enhance the biosensor performances always using the same transduction principle.

The first possibility is to test different biorecognition elements: while Abs have been often preferred,^[^ ^61^, ^62^, ^63^, ^64^, ^65^, ^66^, ^67^, ^68^ ^]^ a non‐Ab scaffold protein^[^ ^60^ ^]^ as well as a DNA aptamer^[^ ^58^, ^59^ ^]^ were also employed to detect IL‐8 and IL‐6, respectively. In the former case, the capture protein based on the cystatin scaffold guaranteed high selectivity, a remarkably low LOD (90 fg mL^−1^, ≈8 × 10^−15^ m) and wide dynamic range spanning six orders of magnitude.^[^ ^60^ ^]^

A second strategy consists in enhancing the active surface area of the working electrode through functionalization with nanomaterials, as in the case of electrochemical signal amplification using gold nanoparticles (AuNPs) on glassy carbon or gold electrode, followed by immobilization of the anti‐IL‐6 DNA oligonucleotide as specific probe.^[^ ^58^, ^59^ ^]^ Other working electrode modification protocols were developed to functionalize indium tin oxide (ITO) electrodes with a layer serving as a primer for the subsequent immobilization of Abs. To this end, ITO electrodes have been modified with 6‐phosphonoexhanoic acid or with epoxy‐substituted polythiophene polymer to further immobilize anti‐IL‐8^[^ ^61^ ^]^ and anti‐ IL1*α* ^[^ ^62^ ^]^ Abs, respectively. In both cases, the modified ITO surfaces were used as working electrodes in impedimetric detection with remarkably low LODs (6 and 3.4 fg mL^−1^, respectively).

**Figure 4:**
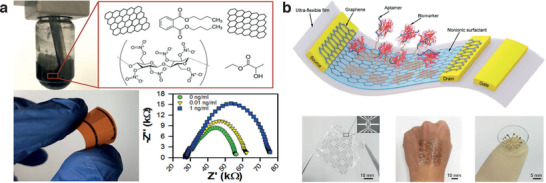
Electrochemical‐ and field effect transistor‐based biosensors for cytokine detection. a) Aerosol‐jet printed graphene immunosensors for IFN and IL‐10. Reproduced with permission.^[^ ^69^ ^]^ Copyright 2020, American Chemical Society. b) Ultraflexible and stretchable graphene‐FET nanosensor for TNF‐*α* detection. Reproduced with permission.^[^ ^83^ ^]^ Copyright 2019, Wiley.

A third way to modulate the device response, and in particular its sensitivity, is to use dedicated materials for signal amplification. This strategy was pursued to enhance the conductivity of ITO electrodes in two different ways. In one case, by first electrodepositing a reduced graphene oxide layer (rGO) followed by immobilization of AuNPs, which served as a substrate for anti‐TNF‐*α* Abs.^[^ ^64^ ^]^ Alternatively, through modification with carbon black^[^ ^63^ ^]^ mixed with polyvinylidene fluoride and poly(glycidyl methacrylate) (LP(GMA)), yielding the combined effect of both increasing the electrochemical signal and providing, through the epoxy groups of GMA, a tethering point for anti‐IL1*α* Abs immobilization. Along the same line, an increase in conductivity of graphene electrodes on a paper substrate was achieved by deposition of polyaniline, whose amino groups have been further used to immobilize anti‐INF‐*γ* Abs.^[^ ^66^ ^]^ Importantly, recent advances in impedimetric detection of cytokines were aimed at demonstrating devices fabricated by high‐throughput methods, such as aerosol‐jet printed graphene immunosensors for interferon‐*γ* (IFN *γ)* and IL‐10^[^ ^69^ ^]^ or screen‐printed gold electrodes for TNF‐*α* detection (**Figure** **4**).^[^ ^70^ ^]^

Besides EIS, other electrochemical methods are amenable for inflammatory biomarker detection. Differential pulse voltammetry (DPV) is a viable alternative: compared to EIS, it allows to focus only on faradic current contribution, and it does not require data fitting with equivalent circuit, apart from enabling more rapid data acquisition. DPV has been employed for detection of IL‐8 upon immobilization of the corresponding Abs on a AuNPs‐rGO composite, which resulted in a reusable and stable (up to three months) device.^[^ ^71^ ^]^ DPV was used also as transduction strategy by Russel et al.^[^ ^72^ ^]^ to sense IL‐6, but this time needle‐shaped microelectrodes were used. Interestingly, at variance with what is typically observed with macro electrodes, the DPV signal was found to increase with increasing cytokine levels.

As an alternative to immobilization of recognition element on the working electrode, Arya et al.^[^ ^73^ ^]^ implemented a so‐called off‐surface 2D polycarbonate membrane matrix functionalized with anti‐TNF‐*α* Abs and located in close proximity of the sensor surface. Sensing was not achieved within a label‐free scheme though but rather as an enzyme labeled immunosensor, namely by further exploiting a sandwiched assay involving secondary Abs and alkaline phosphatase to indirectly quantify the target using DPV.

An elegant solution to achieve electrochemical sensing of cytokines is to use aptamers bearing a redox reporter at one end and exploiting changes in the aptamer structure triggered by binding to the analyte to monitor a concentration‐dependent change (typically, a decrease) in the electron transfer between the redox probe and the electrode. This strategy was pursued, for example, to demonstrate IFN−*γ* and VEGF detection using methylene blue^[^ ^74^ ^]^ and ferrocene^[^ ^75^ ^]^ as redox probes, respectively.

As a general remark, these examples of electrochemical detection of inflammatory biomarkers operate across several orders of magnitude spanning the cytokines clinically relevant range and exhibit low limits of detection. Most notably, apart from validation in buffered test solutions, most of the above‐presented papers reported on electrochemical detection of inflammatory biomarkers in more complex solutions, such as in artificial sweat^[^ ^59^ ^]^ or even in real biological samples such as saliva,^[^ ^61^, ^63^, ^65^, ^71^ ^]^ tears,^[^ ^70^ ^]^ and serum.^[^ ^60^, ^61^, ^62^, ^64^, ^68^ ^]^ As for the response time, electrochemical biosensors for inflammatory biomarkers typically exhibited rapid (within a few minutes) response, as in the case of DPV^[^ ^71^ ^]^ and EIS‐based^[^ ^60^ ^]^ IL‐8 sensors described above which guarantee a response time as low as 9 and 15 min, respectively.

One notable example toward multiplexed detection of cytokines with electrochemical methods involved the use of electrically addressable, diazonium modified Abs that could be immobilized in a controlled way onto multiple Au working electrodes of a fully integrated platform to sense IL‐10 and IL‐1b via EIS measurements.^[^ ^76^ ^]^ Recently, Shen et al. expanded their redox probe tagged electrochemical aptasensor, described above, into a multiplexed biosensor for the simultaneous detection of three cytokines (VEGF, IFN‐*γ*, and TNF‐*α*) by immobilizing different aptamers, each bearing a different redox reporter, on a single working electrode. Changes on the aptamer conformation were transformed into an electrochemical signal due to reduced electron transfer efficiency between the three redox tags and the electrode, following analyte binding to the aptamer and unfolding of the hairpin structure of the latter.^[^ ^77^ ^]^

### 2.3 Field Effect Transistor (FET) Biosensors

In this subsection, we briefly highlight some of the most recently demonstrated label free cytokine biosensors based on FET devices. We here focus on architectures that could not be labeled as electrolyte gated organic transistors (EGOTs), that we thoroughly review in the next sections. The transduction in FET‐based biosensors is typically based on a change in the drain current upon binding of the target analyte to a specific biorecognition element immobilized either on the active material bridging source and drain or (less often) on the gate electrode. One can further classify FET‐based cytokine biosensors with respect to the active material they employ: within this respect, nanotubes/nanowires and 2D atomically layered materials are among the most innovative.

In the former case, real time sensing has been demonstrated for IL6 with (liquid gated) single‐walled carbon nanotubes (CNT) FETs featuring either aptamer^[^ ^78^ ^]^ or Abs,^[^ ^79^ ^]^ invariably immobilized on the CNTs bridging source and drain using 1‐pyrenebutanoic acid succinimidyl ester as linker and by monitoring drain current changes over time upon incubation with IL6‐containing solutions. Alternatively, anti‐TNF‐*α* Abs were immobilized on the floating gates of a CNT‐FET, and the binding of the cytokine to the gate would induce a field effect on the underlying CNTs.^[^ ^80^ ^]^ Silicon nanowires (NWs) have also been employed in NW‐FETcoupled with immunopolymerase chain reaction,^[^ ^81^ ^],^ i.e., a sandwiched assay using anti‐IL2 Abs followed by polymerase chain reaction (PCR) amplification, the latter process detected by the NW‐FET as a pH change that could be related to the IL2 levels in solution.

As for devices based on 2D atomically layered materials, notable examples include TNF‐*α* detection with graphene FET on flexible^[^ ^82^ ^]^ and stretchable^[^ ^83^ ^]^ substrates and real time detection of the same cytokine with a MoS_2_ FET.^[^ ^84^ ^]^

The ion‐sensitive field‐effect transistor architecture has also been applied to demonstrate IL2 and IL4 biosensors with top‐down fabricated nanoscale devices exhibiting a threshold voltage shift upon cytokine binding to the corresponding Abs.

### 2.4 Piezoelectric Biosensors

The transduction principle of these acoustic sensors, which are devices that are based on the presence of a vibrating element, is the change in the resonance frequency of a piezoelectric component when additional mass is attached to it, typically as a consequence of (bio)molecular interaction, such as the binding event between freely diffusing analyte and the biorecognition element that is immobilized on the piezoelectric element. The main transducers are quartz crystal microbalances (QCM) and microcantilevers.^[^ ^34^ ^]^ The application of this technique to cytokine detection has been previously reviewed.^[^ ^85^ ^]^ Bahk et al. improved the sensitivity of the device by coupling the QCM with magnetic beads in an immunosandwich‐like manner, obtaining a linear dynamic range of detection of 40 ng mL^−1^–2 µg mL^−1^ of TNF‐*α*, and relatively short assay time (≈40 min).^[^ ^86^ ^]^ A recent study used the same strategy to increase the sensitivity of the technique to detect TNF‐*α*, reaching a LOD of 1.62 pg mL^−1^, and a range of detection spanning 1–1000 pg mL^−1^, benchmarking their response to the corresponding results obtained by ELISA. However, this QCM‐based biosensor has a long performance time (>4 h) and uses magnetic beads to amplify the signal, which could be considered a kind of label.^[^ ^87^ ^]^

### 2.5 The Road toward Implementation at the Point of Care

ELISA and flow cytometry (e.g., Luminex) are standardized and well‐established methods widely used for clinical detection and quantification of cytokines. They exhibit high selectivity and sensitivity toward the relevant biomarker, even in complex fluids such as human serum or cell culture media, and high reliability and reproducibility—all of which are essential for clinical diagnosis. Both methods still require sample processing (label preparation and incubation), specialized labor, and a significant amount of time, although in recent years, there has been some progress in making these technologies automatic and fast. There are various requirements for a new biosensor technology to be translated from the laboratory bench to clinical use—and compete with the “gold standard” methods. The technology should be amenable for large scale production, possibly cost‐effective and based on materials with long‐term storage stability. The biosensor needs to be reliable and robust, two essential requirements for clinical tools. Selectivity and sensitivity are also important features, especially when considering cytokine detection, due to their low concentrations in body fluids and the sample complexity. Eventually, the biosensor system should be miniaturized/portable, avoid complex measurement setups, and thus be easy to use for nonspecialized practitioners. These characteristics are essential not only for making a biosensor available as a clinical tool in hospitals but also as point‐of‐care devices for home application or in field‐deployment.

While the biosensor technologies described above have proven to be innovative and effective, they each exhibit several limitations hindering their usage as clinical tools. Optical sensors have been implemented with microfluidics and novel fabrication methods to achieve miniaturized devices. However, optical sensor limitations are mostly related to the bulky instruments needed for signal readout. For some optical techniques such as LSPR, the requirement for nanometer‐scale features poses a major hurdle. Piezoelectric‐based biosensors on the other hand require relatively long times for detection. Additionally, despite being in principle a label‐free approach, the sensitivity of piezoelectric‐based sensors has been improved by the use of magnetic beads as labels, which then entail all the complications associated with labeled sensing.

## 3 Electrolyte‐Gated Organic Electronic Transistors

Organic Bioelectronics is rapidly emerging as a leading technique for biosensing. Organic devices comprising organic molecules or polymers as active material enable direct communication between electronics and the biological world.^[^ ^88^ ^]^ Electronically and/or ionically conducting organic molecules and polymers exhibit several advantages compared to their inorganic counterparts: low temperature processability, flexible substrate compatibility, large‐surface area production, easy microfabrication methods,^[^ ^89^ ^]^ and above all, strong ion‐pi noncovalent interactions. Some technologies based on organic electronics are already in commercial use, such as organic light emitted diodes (OLEDs), while most other stand at the forefront of innovative research activities. One device platform is organic transistors, which have proven to be uniquely amenable to a wide variety of bioelectronics applications^[^ ^90^ ^]^ spanning the gamut from neural interfaces^[^ ^91^ ^]^ to electronically functionalized plants.^[^ ^92^ ^]^ In particular, organic electronic transistors represent a versatile and high‐performing platform for the development of novel biosensors.

Organic transistors, like many of their inorganic counterparts, are devices able to amplify a small variation of potential into a larger change in current. They are comprised of three electrodes, generally referred to as source (S), drain (D), and gate (G), using the terminology of the FET. An organic semiconductor (OSC) film connects S and D, forming the transistor channel, and G is connected to the channel through a dielectric material (as in conventional FETs). In the case of EGOTs, an electrolyte serves as the dielectric between G and the OSC, blocking electronic charge transport but allowing ions to move freely. Depending on how ions interact with the OSC, we can define two different kinds of EGOTs: organic electrochemical transistors (OECTs)^[^ ^93^, ^94^, ^95^, ^96^, ^97^ ^]^ and electrolyte gated organic field effect transistors (EGOFETs).^[^ ^98^, ^99^, ^100^ ^]^ In the following subsections, insight into OECT and EGOFET working principles will be given, respectively. The device physics of EGOTs, their architectures, as well as a survey on the different functionalization strategies for biosensing purposes have been discussed in several reviews^[^ ^101^, ^102^, ^103^, ^104^, ^105^, ^106^ ^]^ and the reader can refer to them for more thorough overviews of the field.

### 3.1 OECT Device Physics and Working Principle

**Figure 5:**
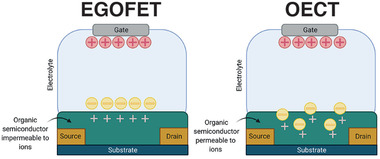
Electrolyte‐gated organic transistors. Left) Schematic representation of electrolyte gated field‐effect transistor (EGOFET), where the organic semiconductor is impermeable to ions and an electrical double layer is created at the semiconductor/electrolyte interface. Right) Schematic representation of an organic electrochemical transistor (OECT), where the organic semiconductor is permeable to ions and results in a volumetric capacitance.

OECTs operate by *V*_GS_‐driven changes in the bulk of the ion‐permeable OSC channel. *V*_GS_ drives ions from the electrolyte into the permeable polymer, thereby modulating its conductivity by (de)doping and thus affecting the current flow (*I*_DS_) (**Figure** **5**). In the early 1980s, Wrighton et al. developed the first OECT, using polypyrrole as channel material.^[^ ^93^ ^]^ The OECT concept lay dormant for some time until Andersson et al. demonstrated^[^ ^94^ ^]^ the first OECTs based on the then‐new material poly(3,4‐ethylenedioxythiophene) doped with the polyanion polystyrene sulfonate (PEDOT:PSS).^[^ ^107^ ^]^ This demonstration foreshadowed some of the utility and versatility of OECTs as even at this early stage, the devices were flexible and printed on paper. Since then, OECTs—now almost ubiquitously based on PEDOT:PSS or similar substances—have become a mainstay of organic electronics, with major efforts spanning from fundamental studies of underlying mechanisms,^[^ ^96^, ^108^, ^109^ ^]^ to novel materials, to a wide array of applications in electronics and bioelectronics.^[^ ^90^, ^97^ ^]^ Recent efforts have also focused on expanding the OSC repertoire used in OECTs. As the majority of OECTs have been based on the p‐type polymer PEDOT:PSS, they are classed as depletion mode devices, i.e., increasing *V*_GS_ depletes the channel of mobile charges and decreases *I*_DS_. Stable n‐type OSCs have been trickier to develop, but recent development with, e.g., poly(benzimidazobenzophenanthroline)^[^ ^110^ ^]^ or naphthalene‐1,4,5,8‐tetracarboxylic diimide with bithiophene^[^ ^111^ ^]^ allow for OECT operation in accumulation mode, i.e., increasing *V*_GS_ increases the mobile charges in the channel and thereby increases *I*_DS_. OECTs can be described by the Bernards and Malliaras model, which relies on their similarity with the metal‐oxide‐semiconductor field effect transistor.^[^ ^96^ ^]^ In this model, the channel current in the steady state behavior is described as follows for the linear and saturation regimes, respectively

For the linear regime (*V*_DS_ higher than *V*_GS_ − *V*_T_)

(1) $$I_{\mathrm{DS}}=\;\mu C^{*}\frac{Wd}{L}\left(1-\frac{V_{\mathrm{GS}}-1/2V_{\mathrm{DS}}}{V_{T}}\right)V_{\mathrm{DS}}$$

For the saturation regime (*V*_DS_ lower than *V*_GS_ − *V*_T_)

(2) $$I_{\mathrm{DS}}=-\mu C^{*}\;\frac{Wd}{L}\frac{{\left(V_{\mathrm{GS}}-V_{T}\right)}^{2}}{2V_{T}}$$

where *I*_DS_ is the current in the channel, *μ* is the charge mobility in the semiconductor, and C^*^ the volumetric capacitance. *W, d*, and *L* are the width, thickness, and length of the channel, *V*_GS_ is the gate voltage, *V*_DS_ is the drain voltage, and *V*_T_ is the threshold voltage. The transconductance *g*_m_ is an important parameter to take into consideration when optimizing OECT performances. *g*_m_ is the derivative of the current in the channel with respect to the gate voltage and can be calculated from Equations (1) and (2). In contrast to FETs, in OECTs *g*_m_ scales with all the geometrical factors, including the thickness of the channel.^[^ ^112^, ^113^ ^]^ This behavior arises from the fact that ionic charges accumulate not only at the semiconductor interface but also penetrating the bulk. Charge mobility *μ* and capacitance C^*^ are also material properties that can be addressed in order to design and optimize the device.^[^ ^114^ ^]^ OECTs can be operated either in nonfaradic or faradic mode. The former is based on capacitive charging of an ionic double layer at the gate that results in the access of ions of opposite sign in the bulk of the OSC, changing its conductivity. On the contrary, in the faradic regime, reduction or oxidation processes at the gate result in channel (de)doping.^[^ ^115^ ^]^

A widely adopted model to account for the detection mechanism of an OECT based biosensors in faradic mode was proposed by Bernards and Malliaras.^[^ ^116^ ^]^ The model relies on potential changes at the electrode/electrolyte interface, due to faradic reactions, thus shifting the effective gate voltage and the current in the channel. OECTs have largely been preferred as amperometric biosensors operated in the faradic regime. In the few examples of OECTs biosensors in the nonfaradic mode, the transduction mechanism was ascribed to charge variations at the surface following analyte binding^[^ ^117^ ^]^ or to limited access of ions to the OSC resulting from their interaction with a peptide‐containing membrane.^[^ ^118^ ^]^

### 3.2 EGOFET Device Physics and Working Principle

EGOFETs feature OSCs that are (in principle) impermeable to ions. Thus, an applied potential between G and S (*V*_GS_) results in ions accumulating from the electrolyte both at the gate/electrolyte and the semiconductor/electrolyte interfaces, forming high‐capacitance electrical double layers (EDLs) that are capacitively coupled. If a potential difference is applied between S and D (*V*_DS_), the field‐induced charge carriers accumulated in the OSC will result in a current flow (*I*_DS_) analogous to inorganic FETs (Figure 5). A water‐gated organic field effect transistor was first demonstrated in 2010 by Kergoat et al. using rubrene and poly(3‐hexylthiophene) (P3HT) as the OSC.^[^ ^98^ ^]^ The channel current in EGOFETs can also be described by Equations (1) and (2) for the OECT model, with the main difference concerning the capacitance *C* and the thickness *d*. If for OECTs the capacitance is described as volumetric capacitance (C^*^), in the EGOFET ionic charges from the electrolyte are usually depicted as accumulating at the semiconductor/electrolyte interface, yielding a capacitance which is independent from the channel thickness.

The EGOFET detection mechanism relies on changes of the EDL at the interface between the electrolyte and the surface (gate electrode or OSC) bearing the biorecognition unit (e.g., antibody, aptamer): upon binding of the analyte, the surface electrochemical potential and/or the EDL capacitance is affected. In EGOFET biosensors the biorecognition element is most often bound to the gate electrode and the recognition with the analyte, thanks to the capacitive coupling, results in a change of the potential drop at the OSC/electrolyte interface affecting the current in the channel; EGOFETs can therefore be seen as potentiometric biosensors.^[^ ^119^ ^]^ One of the main issues in the operation of EGOFETs (and of FET devices in general) as biosensors is the Debye length, which is the length scale at which mobile ions screen the surface charge and is defined as

(3) $$\lambda \;=\sqrt{\frac{\varepsilon \varepsilon_{0}K_{B}T}{2IN_{A}e^{2}}}\;$$

where *ɛ* is the dielectric constant, *ɛ*_0_ is the vacuum permittivity, *K*_B_ is the Boltzmann constant, *T* is the temperature, *I* is the ionic strength of the electrolyte, *N*_A_ is the Avogadro constant, and *e* is the elementary charge.^[^ ^120^ ^]^ In this respect, various strategies have been proposed to deal with the issue of the Debye screening length in FET‐based sensors. Earlier work suggested operating the FET sensors in buffered solutions yielding carefully controlled Debye length that must be large enough to ensure sensing at the device interface but should not be too large as charged unbound species would not be screened and would contribute to nonspecific response.^[^ ^121^ ^]^ This strategy, though, might not always be viable with complex samples, e.g., human bodily fluids, that contain biomacromolecules for which structural integrity and retention of activity relies on relatively high ion concentration.^[^ ^105^ ^]^ Other strategies have been thoroughly reviewed in recent works^[^ ^105^, ^122^ ^]^ and include: modulation of the charged surface morphology; redesign of the measurement scheme, e.g., operation of the FET devices using alternating current signals rather than potentiometric direct current operation; including polyelectrolyte multilayer films in the surface functionalization scheme;^[^ ^123^ ^]^ using smaller biorecognition units, e.g., aptamers instead of antibodies, to ensure that the biorecognition event takes place within the Debye screening length.^[^ ^105^, ^122^ ^]^ With respect to this last solution, Nakatsuka et al. have exploited aptamer folding to not only overcome the Debye screening length issue but also to aid the detection of uncharged targets.^[^ ^124^ ^]^ Most notably, detection beyond the Debye length has been demonstrated by Palazzo et al.^[^ ^120^ ^]^ for an EGOFET biosensor with a mainly capacitive mechanism of sensing that is based on the formation of a Donnan equilibrium that yields an additional capacitance, the effect of which does not depend on the position where such equilibrium is set. In general, although solutions to the issue of detection within or beyond the Debye screening length have been designed, the molecular mechanisms that enable detection even in high ionic strength solutions and at relatively large distance from a charged surface are not fully understood and this hinders the rational design of novel biosensors.

## 4 Electrolyte‐Gated Organic Transistors as Biosensors

Compared to other detection technologies, organic transistors represent an optimal biosensor platform due to their unique features. Organic transistors are based on OSCs, which can be stable in liquid/aqueous environments, including physiological media and bodily fluids.^[^ ^97^ ^]^ They operate at low voltages (0.1–1 V) and therefore require relatively low power (micro‐ to mJ).^[^ ^125^ ^]^ Moreover, they can be fabricated onto inexpensive and disposable substrates such as paper^[^ ^126^ ^]^ or plastic^[^ ^127^ ^]^ and with low‐cost fabrication techniques (spin‐coating, spray coating, screen printing, and inkjet‐printing), facilitating scaling up of their manufacturing. Organic transistors can be miniaturized and integrated with microfluidics, making portable bioelectronic devices relatively straightforward to design and prototype. Interestingly, their fundamental operational principle relies on ion motion, which is also the physiological mechanism for communication, making them effectively suitable for bridging electronics to biology.^[^ ^88^ ^]^

In the last decade, EGOTs, both in OECT and EGOFET configurations, have been extensively used as biosensors. Such applications are reviewed in refs. ^[^ ^88^, ^90^ ^]^, and ^[^ ^129^ ^]^. Here, we focus exclusively on their use for sensing inflammatory biomarkers—or soluble proteins of comparable size, as they pose similar challenges to sensor design and could therefore be readily adapted to sense cytokines or other inflammation‐related biomacromolecules.

### 4.1 OECT‐Based Sensors

**Figure 6:**
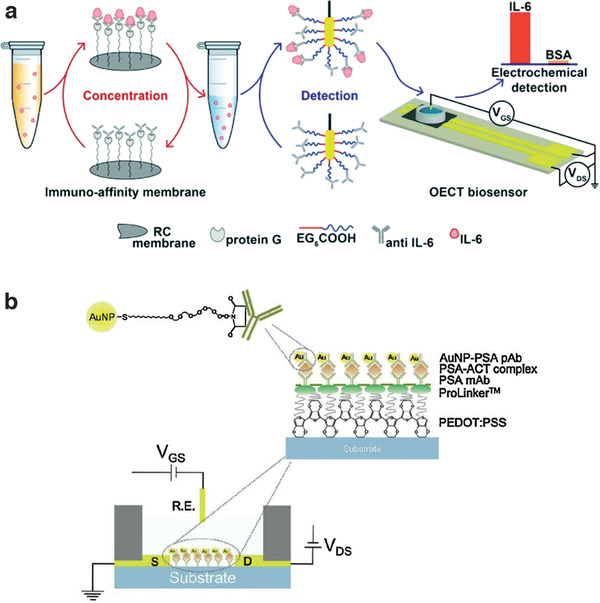
OECT for protein detection. a) PEDOT:PSS based OECT for detection of IL‐6, integrated with an immune‐affinity membrane incubation steps to increase the analyte concentration. Reproduced with permission.^[^ ^144^ ^]^ Copyright 2018, Royal Society of Chemistry. b) OECT immunosensor for detection of PSA with gold particle signal amplification. Reproduced with permission.^[^ ^117^ ^]^ Copyright 2010, Elsevier.

OECT‐based sensors have mostly been used to record electrophysiological signals (e.g., electroencephalogram and electrocardiogram)^[^ ^130^, ^131^, ^132^ ^]^ or for detection of metabolites through enzymatic reactions.^[^ ^111^, ^133^, ^134^, ^135^, ^136^, ^137^, ^138^, ^139^ ^]^ Nevertheless, nonenzymatic detection with OECTs, as in the case of immunobiosensors, has been demonstrated. To this end, Abs,^[^ ^117^ ^]^ aptamers,^[^ ^140^ ^]^ and molecularly imprinted polymers (MIPs)^[^ ^141^ ^]^ have been used as specific biorecognition elements (**Table** **1**). One of the first attempts to develop an Ab‐based biosensor exploiting the OECT architecture was demonstrated by Kim et al., reporting the detection of prostate specific agent (PSA) in complex with the inflammation biomarker 1‐antichymotrypsin (ACT) with a sandwich‐type structure^[^ ^117^ ^]^ (**Figure** **6**b). Monoclonal anti‐PSA Abs (PSA mAbs) were immobilized on the PEDOT:PSS channel, which was functionalized with covalently bound crown‐ether‐terminated linkers. The crown‐ethers provided tight host–guest interactions with the surface‐exposed amine groups of PSA mAbs. Binding of PSA‐ACT complexes to the mAbs‐functionalized channel led to a [PSA‐ACT]‐dependent increase of the drain current that the authors rationalized in terms of an increase of the negative surface charge, due to the PSA isoelectric point of 6.9. The increased negative surface charge, in turn, decreased the dedoping effect of the gate voltage and hence lowered the charge‐carrier depletion. Further sandwiching of the mAb‐bound PSA‐ACT complex with polyclonal anti‐PSA Abs conjugated with Au nanoparticles improved the biosensor performance, yielding both a wider dynamic range and lower PSA LOD down to 1 pg mL^−1^ (Figure 5b).

**Table 1:** A selection of OECT and EGOFET protein sensing demonstrations and their various mechanisms and materials

Target molecule	Functionalized interface	Sensing unit	Channel material	Ref.
**OECT for protein detection**				
Prostate specific agent (PSA)	Channel	Antibody/AuNPs	PEDOT:PSS	[117]
Human epidermal growth factor receptor 2 (HER2)	Gate electrode	Nanoprobes	PEDOT:PSS	[142]
Immunoglobulin‐G (IgG)	Gate electrode	Antibody	PEDOT:PSS	[143]
Interleukin 6 (IL6)	Gate electrode	Antibody	PEDOT:PSS	[144]
l‐histidine, l‐tryptophan	Gate electrode	MIPs	PEDOT:PSS	[147, 148]
*β*‐Amyloids	Channel	Nanoporous membrane	PEDOT:PSS	[118]
Spike proteins (SARS)	Gate electrode	Nanobody	p(g0T2‐g6T2)	[149]
Caspase‐3	Gate electrode	Peptide	PEDOT:PSS	[150]
**EGOFET for protein detection**				
Interleukin 4 (IL‐4)	Gate electrode	Antibody	Pentacene	[156]
Interleukin 6 (IL‐6)	Gate electrode	Antibody/aptamer	Pentacene	[151]
Tumor necrosis factor‐*α* (TNF‐*α*)	Gate electrode	Antibody	Pentacene	[157]
Tumor necrosis factor‐*α* (TNF‐*α*)	Gate electrode	Aptamer	Pentacene	[152]
C‐reactive protein (CRP)	Gate electrode/SAM	Antibody	P3HT	[159]
C‐reactive protein (CRP)	Channel	Antibody	P3HT	[120, 154]
Procalcitonin (PCT)	Channel	Antibody	P3HT	[155]
Vascular endothelial growth factor (VEGF)	Gate electrode	Aptamer hydrogel	DPP‐DTT	[153]
Ricin	Floating gate	Aptamer	P3HT	[174]

Sandwiched modification of the sensing interface was also applied by Fu et al. to demonstrate an OECT‐based biosensor for human epidermal growth factor receptor 2 (HER2), a breast cancer treatment biomarker.^[^ ^142^ ^]^ Despite being an immunosensor, the sensing principle in this case relied on faradic operation of the OECT with the aim of enhancing sensitivity with respect to nonfaradic mode. Nonfaradic mode corresponds to operation of the OECTs as a potentiometric FET governed by capacitive coupling. Here, monoclonal anti‐HER2 Abs was immobilized on the gate. When HER2 was captured by this surface‐bound recognition layer, it promoted further binding of gold nanoparticles (AuNPs) with both anti‐HER2 Abs and horseradish peroxidase (HRP) immobilized on them. The surface concentration of HRP was thus directly proportional to that of HER2. HRP is a redox enzyme that catalyzes oxidation of hydrogen peroxide to water, and in this case led to a faradic decrease of the channel current. The authors interpreted the [HER2]‐dependent response in terms of shift of the effective gate voltage, following the widely used Bernards model,^[^ ^96^ ^]^ and the biosensor performance was remarkable both in terms of LOD (as low as 10^−14^ g mL^−1^) and linear response up to 10^−7^ g mL^−1^.

Ultralow limit of detection (6 × 10^−15^ m) with an OECT‐based biosensor operated in nonfaradic mode was achieved by Macchia et al., who demonstrated an immunoglobulin‐G (IgG) immunosensor by functionalizing the gold gate electrode with specific anti‐IgG Abs.^[^ ^143^ ^]^ The concentration‐dependent change in the channel current occurs in a range from 6 × 10^−15^ to 60 × 10^−12^ m range and was attributed to a shift of the surface potential following the biorecognition process.

To date, and to the best of our knowledge, only two examples of OECT‐based sensors for cytokine detection have been demonstrated. Gentili et al. reported specific IL‐6 detection via recognition by anti‐IL‐6 Abs immobilized on the gate, integrated with an immune‐affinity membrane to pre‐concentrate the analyte in the sample, allowing detection of [IL‐6] as low as 2 ng mL^−1^ (90 × 10^−12^ m).^[^ ^144^ ^]^ IL‐6 binding to the sensing moiety altered the EDL capacitance, affecting channel current modulation by the gate electrode (Figure 6a). Decataldo et al. opted for stepwise functionalization of plasma‐treated PEDOT:PSS channels modified with (3‐Aminopropyl)triethoxysilane (APTES) followed by immobilization of target‐specific Abs using biotin/streptavidin coupling for sensing the growth factor cytokine bone morphogenic protein 2^[^ ^145^ ^]^ down to 1.6 pg mL^−1^.^[^ ^146^ ^]^

Although Abs are by far the most widely exploited biorecognition unit for protein detection, MIPs represent a promising alternative. MIPs can be synthesized with different techniques, but they are invariably characterized by the presence of cavities that are complementary in terms of size, shape, and chemical functionality to the target molecule. The selectivity that can be achieved with MIPs can be extremely high, as demonstrated by Zhang et al., who developed OECT biosensors with electrodeposited MIP‐functionalized gates for chiral recognition of enantiomers of tryptophan and tyrosine ^[^ ^147^ ^]^ and histidine.^[^ ^148^ ^]^ The mechanism behind this amino acid sensing could not be extended to electrochemically inactive molecules as it requires oxidation of the MIP‐allowed substances at the gate, i.e., faradic sensing. Nevertheless, the integration of MIPs at relevant OECT interfaces is viable for detection of larger molecules such as oligo‐ and polypeptides, for which specific recognition by ad hoc synthesized polymers was demonstrated. Along this track, Wustoni et al. demonstrated a MIP‐based OECT biosensor for in vitro detection of A*β* aggregates associated with Alzheimer's disease.^[^ ^118^ ^]^ The working mechanism was based on ion permeability through a nanoporous modified membrane on top of the channel: the membrane was functionalized with molecules able to bind A*β* aggregates and thereby blocking ions from reaching the channel, resulting in a current modulation proportional to the aggregate concentration.

Motivated by the recent pandemic, Guo et al. developed a nanobody‐based OECT for fast and specific detection of SARS‐CoV‐1/2, and Middle East respiratory syndrome coronavirus (MERS‐CoV) antigen spike proteins. The functionalization procedure was performed on the gold gate electrode and followed two steps: a) formation of a self‐assembled monolayer (SAM) and b) incubation with a nanobody‐SpyCatcher fusion protein against spike proteins. The OECT sensor was able to detect the specific antigen in complex media in attomolar concentration range, offering a point‐of‐care platform for virus detection.^[^ ^149^ ^]^ Yu et al. demonstrated an OECT‐based biosensor for the detection of caspase‐3, a proteolytic enzyme involved in apoptotic processes. Their approach was based on measuring the activity of the caspase‐3 on a peptide‐modified gold gate electrode. The detection mechanism relied on surface potential changes related to the amount of peptide bound at the gate electrode. The device was used to study the apoptotic process in HeLa cell culture, providing a facile and sensitive approach for the study of proteolytic enzymes.^[^ ^150^ ^]^

### 4.2 EGOFET‐Based Sensors

EGOFETs have more frequently been preferred for the detection of electrochemically inactive analytes, as is the case of many proteins. As described in the previous section, the detection mechanism underlying EGOFET biosensors involves modification of the EDL at the gate/electrolyte or channel/electrolyte interface upon the specific biorecognition event. Due to well‐established protocols for biomolecule immobilization on bare or modified metal surfaces (primarily gold), the most common approach for biosensing with EGOFETs relies on Ab attachment to a gold gate. The use of aptamers instead of Abs ^[^ ^151^, ^152^, ^153^ ^]^ or the confinement of the biorecognition unit at the channel/electrolyte interface^[^ ^120^, ^154^, ^155^ ^]^ are less common (Table 1).

EGOFETs were first used for sensing cytokines, specifically interleukin 4 (IL‐4), by Casalini et al.^[^ ^156^ ^]^ Two strategies for confinement of anti‐IL‐4 Abs at the Au gate/electrolyte interface were compared: i) covalent attachment of the Abs to a 6‐aminohexanethiol (HSC_6_NH_2_) SAM through glutaraldehyde crosslinking and ii) noncovalent, though selective, interaction of the Abs with a (sub)monolayer of Protein G, bound to the gate via an N‐terminal hexahistidine tag. Although both approaches result into gate‐immobilized anti‐IL‐4 Abs, the second protocol, which should yield more uniformly oriented Abs with respect to the SAM‐based approach, was found to be more effective for cytokine. The difference was ascribed to higher probability of specific binding events, based on single molecule force spectroscopy approaches. Along this line, Diacci et al. demonstrated EGOFET biosensors for a second cytokine, IL‐6, by gating the device with Au electrodes functionalized either with the above described ProteinG/Ab approach, or using a smaller (≈100 amino acid) peptide aptamer bound directly to gold via a polyhistidine tag in a single step approach.^[^ ^151^ ^]^ The two approaches (full Ab and aptamer) likely yielded different surface concentrations of the biorecognition unit as well as binding events taking place at different distances from the Au surface. However, both biosensors exhibit dynamic ranges spanning four orders of magnitude (pm and low‐nm regime) and LODs as low as 1 × 10^−12^ m. These LODs are comparable to serum levels under physiological conditions, meaning that these biosensors could potentially be used to monitor patients suffering from inflammatory response to various pathologies.

**Figure 7:**
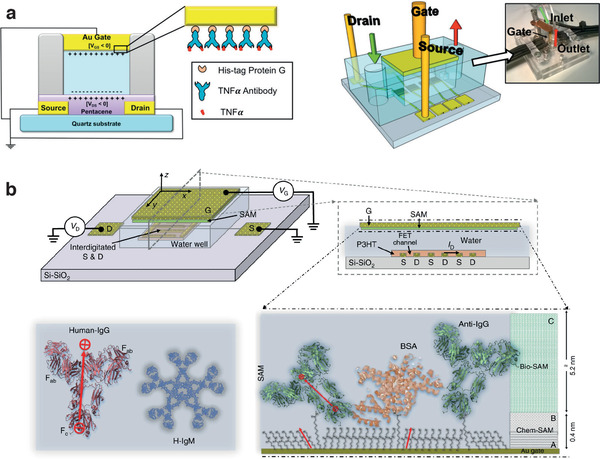
EGOFET for protein detection. a) Microfluidic integrated EGOFET immunosensor for the detection of TNF‐*α*. Reproduced with permission.^[^ ^157^ ^]^ Copyright 2016, American Chemical Society. b) EGOFET based immunosensor for single protein detection. Reproduced with permission.^[^ ^161^ ^]^ Copyright 2018, Springer Nature.

EGOFETs were also demonstrated as biosensors toward another cytokine, TNF‐*α*, by Berto et al., again using either ProteinG‐bound Abs^[^ ^157^ ^]^ or peptide aptamers^[^ ^152^ ^]^ to selectively bind the pleiotropic cytokine. In the first case, the immunosensor was integrated in a polydimethylsiloxane microfluidic system and could detect TNF‐*α* down to 100 × 10^−12^ m (**Figure** **7**a). The operability in more complex samples was assessed by detecting TNF‐*α* released into cell culture medium by human monocytes in vitro. The authors explained the device sensitivity in terms of the density of states (DOS) of the organic semiconductor used (pentacene), ascribing the large variations of the device response to shifts of the Fermi level in the (super‐exponential) tail of the DOS. The later work by Berto et al. reported on a peptide aptasensor for TNF‐*α*, reaching a LOD as low as 1 × 10^−12^ m even in cell culture media containing 10% fetal bovine serum.^[^ ^152^ ^]^ Parkula et al. in 2020 presented a novel lab‐on‐a‐chip integrating a microfluidic system with an EGOFET device. The device features four gate electrodes, three of which are functionalized with a peptide aptamer specific toward TNF‐*α*. The fourth electrode is covered only by a self‐assembly monolayer (SAM) with no biorecognition elements, thus serving as an internal control on potentially interfering nonspecific binding and/or on current changes caused by external factors, such as bias stress. This setup provides measurements in triplicate, thus enabling a check on the reproducibility of the biosensor response. Moreover, the EGOFET shows high selectivity and a detection limit as low as 3 × 10^−12^ m.^[^ ^158^ ^]^ This example shows how EGOFET biosensors are suitable for miniaturization and integration with microfluidic system.

The biorecognition moiety can also be confined at the interface between the organic semiconductor and the electrolyte, to sense protein targets freely diffusing in solution as demonstrated by Magliulo et al.,^[^ ^154^ ^]^ Palazzo et al.,^[^ ^120^ ^]^ and Seshadri et al.^[^ ^155^ ^]^ In the former work, anti‐CRP Abs were physisorbed on a P3HT film bridging source and drain and a nonionic hydrophilic polymer (*N*‐[tris(hydroxy‐ methyl)methyl]acrylamide‐lipoic acid conjugate, pTHMMAA) was used as a blocking agent to minimize nonspecific adsorption on the device channel. Binding of CRP to the corresponding Abs at the P3HT surface caused a decrease of the drain current that was tentatively ascribed to changes in the gating capacitance upon analyte immobilization at the channel/electrolyte interface. Simplicity and time‐effectiveness of the functionalization strategy were among the main features of the proposed device, which reached a LOD corresponding to 2 × 10^−12^ m was estimated, with the biosensor working in a dynamic range spanning five orders of magnitude.^[^ ^154^ ^]^ This represented a substantial advancement with respect to the phospholipid combined with streptavidin or avidin monolayer cast on P3HT OSC, by the same authors.^[^ ^120^ ^]^ A few years later, a similar strategy was exploited to sense procalcitonin (PCT), which may serve as a sepsis marker, down to the pm regime and spanning physiologically relevant ranges. Anti‐PCT Abs were again physically adsorbed on a P3HT film surface, but instead of choosing pTHMMAA as blocking agent, the authors used bovine serum albumin (BSA). At variance with what was observed for CRP detection, where the transconductance was mostly affected by the biorecognition event, the drain current decrease following PCT binding is mainly contributed by changes in the threshold voltage that could be ascribed to the negative charge of PCT at the operational pH, which would act as trap sites for the (hole) charge carriers.^[^ ^155^ ^]^ Recently, Torsi's group demonstrated EGOFET‐based biosensors reaching the ultimate sensitivity of single molecule detection (hence the acronym SiMoT to identify their device architecture) (Figure 7b). The key ingredient to the ultralow LOD is a millimeter‐sized gold lamina functionalized with a mixed 3‐mercaptopropionic acid (3‐MPA): mercaptoundecanoic acid (MUA) SAM. MUA enables covalent immobilization of trillions of capturing proteins (followed by further blocking with BSA) while MPA forms a H‐bonding network that cooperatively rearranges upon analyte binding, thus causing a shift of the work function of the gate electrode. The remarkable 1(±1) molecules LOD has been achieved for different targets, including CRP^[^ ^159^ ^]^ and IgM and IgG.^[^ ^160^, ^161^ ^]^ Pallu et al. used a different approach by showing a proof of concept for DNA hydrogels to be used for detection of VEGF. The authors chose to introduce a hydrogel to exploit its ability to change its 3D structure upon target binding as an attempt to overcome traditional limitations set by the Debye length in common buffer solutions at the gate/electrolyte interface. The DNA hydrogel could then gate an EGOT and, by incorporating a VEGF‐specific aptamer within the hydrogel, the presence of the growth factor in the electrolyte surrounding the hydrogel could be detected.^[^ ^153^ ^]^

Many of the examples reviewed in the present section are demonstrations of biosensors in buffered spiked solutions of the relevant cytokine. The translation to complex fluids such as plasma, serum, or saliva poses a significant challenge to the design of EGOT‐based biosensors since, in the “traditional” layout, two interfaces are simultaneously exposed to the sample, i.e., both the gate and the OSC film, and therefore both might represent adsorption sites for interfering species. Avoiding nonspecific binding at the sensing interface is particularly challenging for label‐free biosensors. The main strategies used for this purpose are focused on adding blocking agents (e.g., BSA^[^ ^117^, ^142^, ^143^, ^151^, ^152^, ^155^, ^159^ ^]^) or by surface chemistry (e.g., olygoethyleneglycol sub‐monolayers^[^ ^144^, ^154^, ^159^ ^]^), in order to fill the nonfunctionalized spots between the surface‐tethered specific biorecognition elements. A third approach would be sample pre‐treatment, although it is not recommended for biomarkers with low physiological/pathological concentrations.^[^ ^162^, ^163^ ^]^ Extended/floating gate is an elegant and effective solution to the problem^[^ ^164^ ^]^ that avoids the contact between the sample and the OSC, therefore limiting the problem of nonspecific adsorption to the gate surface only and basically deconstructing the traditional EGOT architecture into two parts, namely the transistor and the sensing chamber. In the extended gate configuration, the transistor is separated by a dielectric (ion gel or electrolyte solution in the case of EGOTs) from the first arm of a gate electrode that extends (hence the name) far from it with a second (sensing) arm functionalized with the recognition element that reaches a physically distinct reservoir containing the sample. The potential of the extended gate is not controlled directly (thus the devices are consequently termed as “floating”) by the gate voltage, which is instead imposed between the source and a second electrode, and capacitively coupled to the sensing arm through the sample. Extended gate OFETs were reported for IgG,^[^ ^165^, ^166^ ^]^ IgA,^[^ ^167^ ^]^ human glycoprotein,^[^ ^168^ ^]^ histidine‐rich protein (albumin),^[^ ^169^ ^]^ histamine,^[^ ^170^ ^]^ phosphorprotein,^[^ ^171^ ^]^ CRP,^[^ ^172^ ^]^ and glutathione^[^ ^173^ ^]^ sensing. Other notable examples of applications that provide evidence for effective sensing in real‐life scenarios are CRP sensors integrated with a smart surgical catheter^[^ ^172^ ^]^ and a floating gate EGOT for aptamer‐based detection of ricin in complex food matrices like milk or orange juice.^[^ ^174^ ^]^ Despite falling outside the focus of this paper, it is worth mentioning that EGOFETs have also been demonstrated as biosensors for other protein biomarkers, e.g., antidrug Abs,^[^ ^175^ ^]^ immunoglobulin,^[^ ^160^, ^176^ ^]^ protein aggregates,^[^ ^177^ ^]^ viral proteins,^[^ ^178^, ^179^ ^]^ alpha‐fetoprotein,^[^ ^180^ ^]^ and myelin basic protein.^[^ ^181^ ^]^

## 5 Outlook

Inflammatory processes are driven and regulated by networks of multiple cytokines and different relative levels between these proteins are responsible for different pathological states. Deeper understanding of inflammatory pathologies will thus require development of (ideally miniaturized) sensors able to simultaneously analyze multiple markers in the same sample. In addition to the ability to identify the “cytokine fingerprint” of various pathologies, these sensors will require only minimal sample quantities for the single analysis, rather than collection of multiple samples for individual detection of each cytokine. Multifunctionality will also lead directly to more simple systems at the point‐of‐care as well as lower power requirements, higher‐throughput manufacturing, and likely lower cost to healthcare providers and patients. To date, only a few researchers have taken up the challenge to demonstrate such detection of multiple cytokines.^[^ ^42^, ^76^, ^182^, ^183^, ^184^ ^]^ We believe that OECTs and EGOFETs are suitable platforms to build these next‐generation multifunctional sensors as they can combine optimal LOD, ease of manufacture, and ease of integration with other (bio)electronic systems and therapies.

In regard to therapeutic approaches, inflammatory diseases are traditionally treated by (local or systemic) administration of anti‐inflammatory drugs.^[^ ^185^ ^]^ More recent treatment methods have also used electrical stimulation, primarily of the vagus nerve, to mediate inflammatory responses.^[^ ^186^, ^187^, ^188^ ^]^ Traditional drug delivery methods, such as injections or oral administration, are well known procedures with systemic effects on the body. However, these methods present numerous drawbacks since they act on multiple pathways simultaneously, effect regions outside of the primary inflammation zone, and thus have the potential to cause mild to severe side effects.^[^ ^189^ ^]^ Continuous monitoring of inflammation markers coupled with an automatic drug delivery system may reduce the amount of molecules used and give a personalized treatment to reduce side effects.^[^ ^188^ ^]^ The very same organic electronic materials, methods, and concepts applied in the development of OECTs and EGOFETs have been used for a variety of such drug delivery systems, including controlled release electrodes, electrophoretic transport through ion exchange membranes, and even micromechanical delivery systems.^[^ ^88^, ^190^ ^]^ Likewise, recent developments in “electroceutical” treatment of inflammatory disorders have also turned to organic electronics.^[^ ^191^, ^192^ ^]^ In these systems, electronic signals are delivered locally—most frequently to the vagus nerve—to modulate downstream inflammatory responses.

The parity of fabrication techniques, materials, and operating conditions make the integration of cytokine sensing OECTs and EGOFETs with organic electronic delivery technologies and “electroceuticals” fairly straightforward and thus a promising path toward personalized treatment systems. However, in addition to the challenges of development and optimization of the pharmaceutical and electrical actuators in such systems, the OECT and EGOFET sensors will require standardization and enhanced reliability before they can be implemented at the PoC. This is of course a major focus of the research community. The authors of this Progress Report have recently spearheaded the Marie Skłodowska Curie European Training Network BORGES (Biosensing with Organic Electronics)^[^ ^193^ ^]^ to specifically investigate the underlying mechanisms of OECT and EGOFET sensors with the ultimate aim of providing more standard protocols for their use in clinical settings. The project spans fundamental academic research, to industry expertise in instrumentation and modeling, to pre‐clinical expertise close to the point‐of‐care. Of course, there are many challenges in reaching the aims of the BORGES project, and indeed the goals of the OECT and EGOFET biosensor community in general: miniaturization will require iterated steps of optimization and testing; reliably introducing samples at the point‐of‐care will require further integration with, e.g., fluidic systems; and achieving low‐cost high‐throughput manufacturing will require a great deal of development effort to translate microfabrication techniques to, e.g., printing methods. Ultimately, we aim to provide the scientific and healthcare communities with a suite of sensor technologies that are robust, multifunctional, and certified for use with human patients.

## Conflict of Interest

The authors declare no conflict of interest.
